# Genome-Wide Association Study for Chronic Hepatitis B Infection in the Thai Population

**DOI:** 10.3389/fgene.2022.887121

**Published:** 2022-06-13

**Authors:** Saeideh Ashouri, Seik-Soon Khor, Yuki Hitomi, Hiromi Sawai, Nao Nishida, Masaya Sugiyama, Yosuke Kawai, Nawarat Posuwan, Pisit Tangkijvanich, Piyawat Komolmit, Makoto Tsuiji, Vorasuk Shotelersuk, Yong Poovorawan, Masashi Mizokami, Katsushi Tokunaga

**Affiliations:** ^1^ Genome Medical Science Project, National Center for Global Health and Medicine, Toyama, Tokyo,Japan; ^2^ Department of Human Genetics, Graduate School of Medicine, The University of Tokyo, Tokyo, Japan; ^3^ Department of Microbiology, Hoshi University School of Pharmacy and Pharmaceutical Sciences, Tokyo, Japan; ^4^ Genome Medical Science Project, National Center for Global Health and Medicine, Ichikawa, Chiba, Japan; ^5^ Chulabhorn International College of Medicine, Thammasat University, Rangsit Campus, Pathum Thani, Thailand; ^6^ Center of Excellence in Clinical Virology, Department of Pediatrics, Faculty of Medicine, Chulalongkorn University, Bangkok, Thailand; ^7^ Center of Excellence in Hepatitis and Liver Cancer, Faculty of Medicine, Chulalongkorn University, Bangkok, Thailand; ^8^ Center of Excellence in Liver Diseases, King Chulalongkorn Memorial Hospital, Thai Red Cross Society, Bangkok, Thailand; ^9^ Liver Fibrosis and Cirrhosis Research Unit, Faculty of Medicine, Chulalongkorn University, Bangkok, Thailand; ^10^ Department of Pediatrics, Center of Excellence for Medical Genomics, Faculty of Medicine, Chulalongkorn University, Bangkok, Thailand

**Keywords:** chronic hepatitis B, GWAS, SNP, HLA, allele, haplotype

## Abstract

To identify novel host genetic variants that predispose to hepatitis B virus (HBV) persistence, we performed the first genome-wide association study in the Thai population involving 318 cases of chronic hepatitis B and 309 healthy controls after quality control measures. We detected the genome-wide significant association of the *HLA* class II region (*HLA-DPA1/DPB1*, rs7770370, *p*-value = 7.71 × 10^−10^, OR = 0.49) with HBV chronicity. Subsequent *HLA* allele imputation revealed HLA-DPA1*01:03 (*Pc* = 1.21 × 10^−6^, OR = 0.53), HLA-DPB1*02:01 (*Pc* = 2.17 × 10^−3^, OR = 0.50), and HLA-DQB1*06:09 (*Pc* = 2.17 × 10^−2^, OR = 0.07) as protective alleles, and HLA-DPA1*02:02 (*Pc* = 6.32 × 10^−5^, OR = 1.63), HLA-DPB1*05:01 (*Pc* = 1.13 × 10^−4^, OR = 1.72), HLA-DPB1*13:01 (*Pc* = 4.68 × 10^−2^, OR = 1.60), and HLA-DQB1*03:03 (*Pc* = 1.11 × 10^−3^, OR = 1.84) as risk alleles for HBV persistence. We also detected suggestive associations in the *PLSCR1* (rs35766154), *PDLIM5* (rs62321986), *SGPL1* (rs144998273), and *MGST1* (rs1828682) loci. Among single-nucleotide polymorphisms in the *PLSCR1* locus, rs1061307 was identified as the primary functional variant by *in silico/in vitro* functional analysis. In addition to replicating the association of the *HLA* class II region, we detected novel candidate loci that provide new insights into the pathophysiology of chronic hepatitis B.

## Introduction

Hepatitis B virus (HBV) causes one of the major infectious diseases in humans, and is associated with an increased risk of severe liver complications, such as hepatic decompensation, cirrhosis, and liver cancer (WHO, 2017; Polaris Observatory Collaborators, 2018). Chronic HBV infection which is defined by the presence of hepatitis B surface antigen (HBsAg) in the host for at least 6 months (WHO, 2017), is considered a polygenic and multifactorial condition (Thursz, 2001; Akcay et al., 2018; Podlaha et al., 2019). Of the 292 million people living with chronic HBV infection around the world (Polaris Observatory Collaborators., 2018), 68% reside in the African and Western Pacific regions. Additionally, among individuals who are infected with HBV in adulthood, only 1% to 5% develop chronic HBV infection (Bertoletti and Kennedy, 2015). These observations led to the speculation that the persistence of HBV infection may be dependent on the heterogeneity of the host genetic background.

Numerous studies have been conducted to detect host genetic factors that are associated with HBV persistence. Two twin studies (Lin et al., 1989; Xu et al., 2004) showed significant differences in the concordance rate of infection, clinical phenotype, and serological patterns between monozygotic twins and dizygotic twins, and between monozygotic twins and the control groups. A genome-wide linkage study (Frodsham et al., 2006) and several case-control association studies (Ben-Ari et al., 2003; Deng et al., 2004; Chang et al., 2005; Singh et al., 2007; Qiu et al., 2012; Riazalhosseini et al., 2018) showed the contribution of other genes, especially immunologically relevant genes, such as *HLA*, *CRF2*, *TNFA*, *IFNG*, *CCR5*, *IL-6*, and *ESR*. The first genome-wide association study (GWAS) of chronic hepatitis B (Kamatani et al., 2009) showed the significant association of 11 SNPs in a region including *HLA-DPA1* and *HLA-DPB1*; after more samples were added to the first sample set (Mbarek et al., 2011), an independent association was detected between *HLA-DQ* locus and HBV persistence. Subsequent GWASs in other Asian populations (Nishida et al., 2012; Hu et al., 2013; Kim et al., 2013; Chang et al., 2014; Jiang et al., 2015; Li Y. et al., 2016; Sakaue et al., 2021; Zeng et al., 2021) replicated the significant association of the *HLA* class II region, and showed the association of some novel loci, such as *UBE2L3*, *HLA-C*, *HLA-DQA2*, *HLA-DQB2*, *EHMT2*, *TCF19*, *CFB*, *NOTCH4*, *HLA-C*, *HLA-DO*, *CD40*, *GRHL2*, and *INTS10* (Hu et al., 2013; Kim et al., 2013; Chang et al., 2014; Jiang et al., 2015; Li Y. et al., 2016; Zeng et al., 2021). However, the genetic factors that contribute to the pathogenesis of chronic HBV infection as a complex and heterogenetic disease have not yet been fully identified.

There are more than three million patients with chronic HBV infection in Thailand, and HBsAg seroprevalence rate is 6.42% (Posuwan et al., 2019). Hepatocellular carcinoma associated with HBV infection is one of the major causes of cancer-related deaths in Thailand (Chonprasertsuk and Vilaichone, 2017). Previous case-control association studies have reported the association of several genes, such as *TNFA*, *HLA-DRB1*, *IL-18*, *IL28B*, and *HLA-DP* (Hirankarn et al., 2007; Kummee et al., 2007; Kamatani et al., 2009; Posuwan et al., 2014; Kimkong et al., 2015), with the chronicity of HBV infection in Thailand. To further dissect the genetic architecture of chronic HBV infection in this population, we conducted a GWAS using 627 samples, including 318 cases of chronic HBV infection and 309 healthy controls; in addition to replicating the association of *HLA-DPA1*/*DPB1*, we detected the association of some novel candidate loci.

## Results

### GWAS

We genotyped 647 samples, including 329 cases of chronic HBV infection and 318 healthy controls, using the Affymetrix Axiom Genome-Wide ASI 1 Array (Materials and Methods). We then conducted genotype calling and removed 1 case sample with a genotyping call rate <0.97.

An identical-by-descent (IBD) test was conducted using PLINK (v1.9) (Purcell et al., 2007), and 16 samples with PI_HAT >0.1875, including 9 cases and 7 controls, were excluded from the data. We then performed a principal component analysis (PCA) for detecting possible population outliers or population stratification. PCA revealed that the Thai samples belonged to the Asian population (Supplementary Figure S1A). Three samples, including 1 case and 2 controls, were detected as population outliers, and were excluded from the data. As there are several ethnic groups in Thailand, we also conducted a PCA on Thai samples only, and found that the genetic background of the cases and controls matched (Supplementary Figure S1B). None of the samples had discordant gender information between the clinical data and genetically determined sex. Basic characteristics of samples are provided in Supplementary Table S1.

**FIGURE 1:**
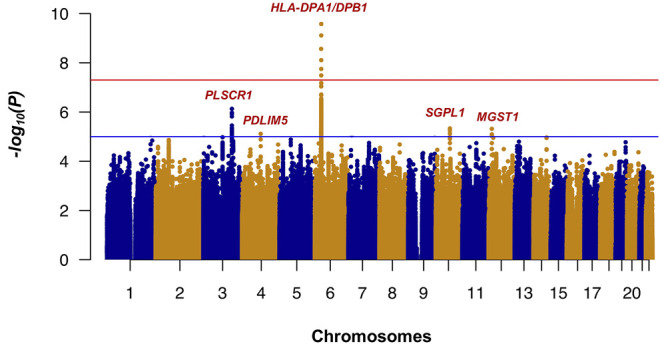
Results of the GWAS (Manhattan plot) for chronic hepatitis B in the Thai population. The *HLA-DPA1/DPB1* exhibited the most significant association with chronic hepatitis B in the Thai population. *p*-values were calculated using the chi-squared test for 318 patients and 309 controls in the GWAS stage. The horizontal red and blue lines show the genome-wide significant threshold (*p*-value <5 × 10^−8^) and suggestive threshold (*p*-value <1 × 10^−5^), respectively.

**FIGURE 2:**
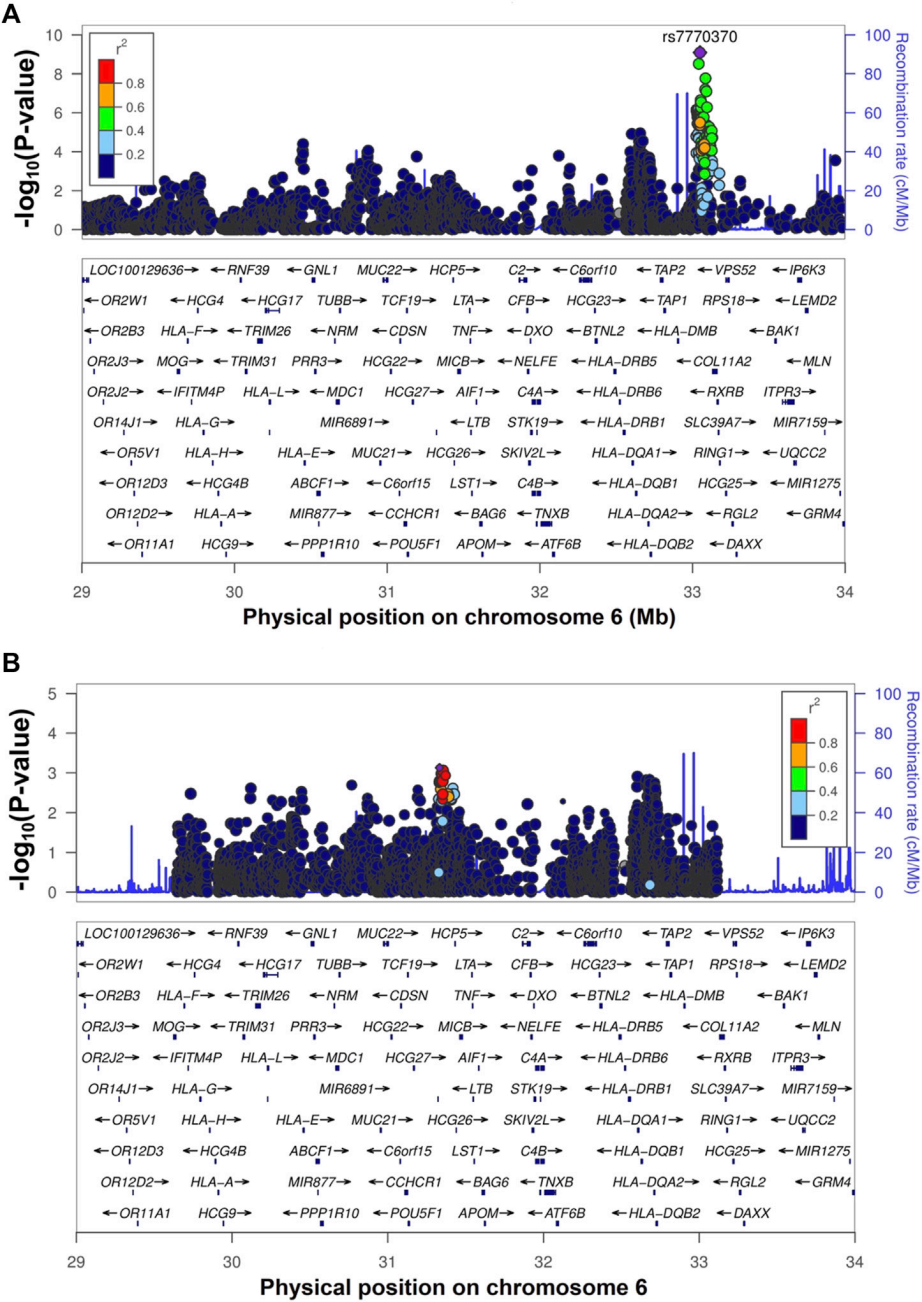
The regional association plot for the *HLA-DPA1/DPB1* region. Figure **(A)** shows the regional association plot of the *HLA* region with the GWAS top SNP (rs7770370) shown by a purple circle. Figure **(B)** shows the plot of the *HLA-DPA1/DPB1* region after conditioning on the top SNP (rs7770370).

Whole-genome imputation was conducted using IMPUTE2 software (v2.3.2) (Howie et al., 2009) with 1,000 Genomes phase 3 as the reference panel. We then applied some post-imputation quality control (QC) to exclude SNPs and short INDELs with a call rate <0.97, minor allele frequency (MAF) < 0.01, and Hardy-Weinberg equilibrium (HWE) test *p*-value <1 × 10^−6^. None of the samples had a genotype calling rate <0.97. The association analysis of whole-genome imputation data, including 318 cases, 309 controls, and 6,317,193 SNPs/short INDELs, was conducted using the 2 × 2 contingency chi-square test (Supplementary Table S2). The genomic inflation factor (λ_GC_) was 1.036 for all tested variants, and after removal of the SNPs/short INDELs of the extended *HLA* region (Hg19: chr6:25,652,429-33,368,333), it decreased to 1.031. The Manhattan plot of the association analysis results is shown in Figure 1. The Q-Q plots of the observed *p*-values against the expected distribution of the association test statistics before and after the exclusion of SNPs from the *HLA* region are shown in Supplementary Figures S2A,S2B, respectively. The most significant association was detected in the *HLA-DPA1/DPB1* region on chromosome 6 (rs7770370, located in the intron of the *HLA-DPB1* gene, *p*-value = 7.71 × 10^−10^, OR = 0.49, 95% CI = 0.39–0.61) (Figure 2A), replicating the association of this region with chronic HBV infection that was reported in previous studies (Kamatani et al., 2009; Nishida et al., 2012; Hu et al., 2013; Kim et al., 2013; Chang et al., 2014). No other locus achieved the genome-wide level of significance (*p*-value < 5 × 10^−8^). As there are highly variable linkage disequilibrium patterns and chromosomal recombination in the *HLA* region (Williams, 2001), we conducted a conditional analysis to check for any other genes or loci in this region that were independently associated with chronic HBV infection, but were masked by the association signal from *HLA-DPA1/DPB1*. We extracted 5,590 SNPs from the *HLA* region, and conducted logistics regression conditioning on the lead SNP (rs7770370). However, no other locus in this region achieved the level of significance after Bonferroni correction (significance threshold: 0.05/5,590 = 8.9 × 10^−6^) (Figure 2B).

Additionally, four other loci (Supplementary Table S2), i.e., rs35766154 located 13 kb 5′ of the *PLSCR1* (Phospholipid Scramblase 1) gene on chromosome 3 (*p*-value = 7.39 × 10^−7^, OR = 0.43, 95% CI = 0.31–0.61), rs62321986 located 28 kb 5’ of the *PDLIM5* (PDZ and LIM Domain 5) gene on chromosome 4 (*p*-value = 7.68 × 10^−6^, OR = 2.58, 95% CI = 1.68–3.96), rs144998273 located in the intron of *SGPL1* (Sphingosine-1-Phosphate Lyase 1) gene on chromosome 10 (*p*-value = 4.64 × 10^−6^, OR = 2.52, 95% CI = 1.67–3.78), and rs1828682 located in the intron of the *MGST1* (Microsomal Glutathione S-Transferase 1) gene on chromosome 12 (*p*-value = 4.82 × 10^−6^, OR = 0.32, 95% CI = 0.19–0.53), achieved the suggestive level of significance (Supplementary Figure S3). We used a TaqMan genotyping assay for experimental validation of the GWAS top SNP (rs7770370) from the *HLA* region, and the top genotyped and the top imputed SNPs on chromosomes 3, 4, 10, and 12 (Supplementary Table S3). All nine SNPs were successfully validated with a mean concordance rate of 98% (94% to 100%).

### Association Analysis of *HLA* Alleles and Haplotypes With Chronic HBV Infection

In an attempt to replicate the reported association of different *HLA* class II alleles and discover novel alleles that predispose to chronic HBV infection in the Thai population, we conducted four-digit *HLA* allele imputation in the class II region (*HLA-DPA1*, *-DPB1*, and *-DQB1*) with the SNP data of all samples using an in-house HIBAG reference panel specific to the Thai population. As post-imputation QC, *HLA* alleles with a posterior probability of <0.5 were excluded from the data. To assess the accuracy of the *HLA* imputation, 300 samples, including 150 cases and 150 controls, were randomly chosen for *HLA* typing. The mean concordance rate between the imputed (after post-imputation QC) and genotyped *HLA-DPA1*, *-DPB1*, and *-DQB1* genotypes was 98%, indicating the high accuracy of our in-house reference. We excluded samples with discordant genotyping information between the two methods, and *HLA* alleles/haplotypes with an allele frequency <0.01 in both the cases and controls. We then performed a case-control association analysis for the remaining *HLA* alleles/haplotypes using Pearson’s chi-squared test. *p*-values were corrected for the number of alleles/haplotypes tested (shown as P-corrected [*Pc*]). An association was considered significant if *Pc* < 0.05. HLA-DPA1*01:03 (*Pc* = 1.21 × 10^−6^, OR = 0.53), HLA-DPB1*02:01 (*Pc* = 2.17 × 10^−3^, OR = 0.50), and HLA-DQB1*06:09 (*Pc* = 2.17 × 10^−2^, OR = 0.07) showed a significant protective effect against chronic HBV infection, while HLA-DPA1*02:02 (*Pc* = 6.32 × 10^−5^, OR = 1.63), HLA-DPB1*05:01 (*Pc* = 1.13 × 10^−4^, OR = 1.72), HLA-DPB1*13:01 (*Pc* = 4.68 × 10^−2^, OR = 1.60), and HLA-DQB1*03:03 (*Pc* = 1.11 × 10^−3^, OR = 1.84) were significantly associated with susceptibility to chronic HBV infection (Supplementary Tables S4–S6).

To determine whether other *HLA* alleles had a significant association with chronic HBV infection that was masked by the most significant allele, we conducted a logistic regression analysis conditioned on the top *HLA* allele (HLA-DPA1*01:03); however, no other allele achieved the threshold level for significance (*p*-value: 0.05/18 = 2.7 × 10^−3^), implying that the primary *HLA* allele that is significantly associated with protection against HBV chronicity in the Thai population is HLA-DPA1*01:03.

We also analyzed the association of different *HLA* haplotypes with chronic HBV infection using Pearson’s chi-squared test. Haplotypes were estimated using the Arlequin algorithm (v3.0) (Excoffier et al., 2007). *HLA* haplotypes with an allele frequency <0.01 in both the cases and controls were excluded. Among two-locus *HLA-DPA1-DPB1* haplotypes (Supplementary Table S7), HLA-DPA1*01:03-DPB1*02:01 (*Pc* = 9.5 × 10^−4^, OR = 0.47) showed the strongest protective association against HBV persistence, and HLA-DPA1*02:02-DPB1*05:01 (*Pc* = 3.74 × 10^−4^, OR = 1.68) showed the strongest association with susceptibility to HBV persistence. Among two-locus *HLA-DPB1-DQB1* haplotypes (Supplementary Table S8), HLA-DPB1*05:01-DQB1*03:03 (*Pc* = 3.08 × 10^−3^, OR = 2.27) and HLA-DPB1*13:01-DQB1*05:02 (*Pc* = 9.22 × 10^−3^, OR = 5.42) were detected as haplotypes that were significantly associated with susceptibility to persistent HBV infection. The only significant three-locus *HLA-DPA1-DPB1-DQB1* haplotype (Supplementary Table S9) was HLA-DPA1*02:02-DPB1*05:01-DQB1*03:03 (*Pc* = 9.68 × 10^−3^, OR = 2.07), which was associated with susceptibility to chronic HBV infection.

### Identification of the Primary Functional Variants in the *PLSCR1* Locus by *in Silico/in vitro* Analysis

**FIGURE 3:**
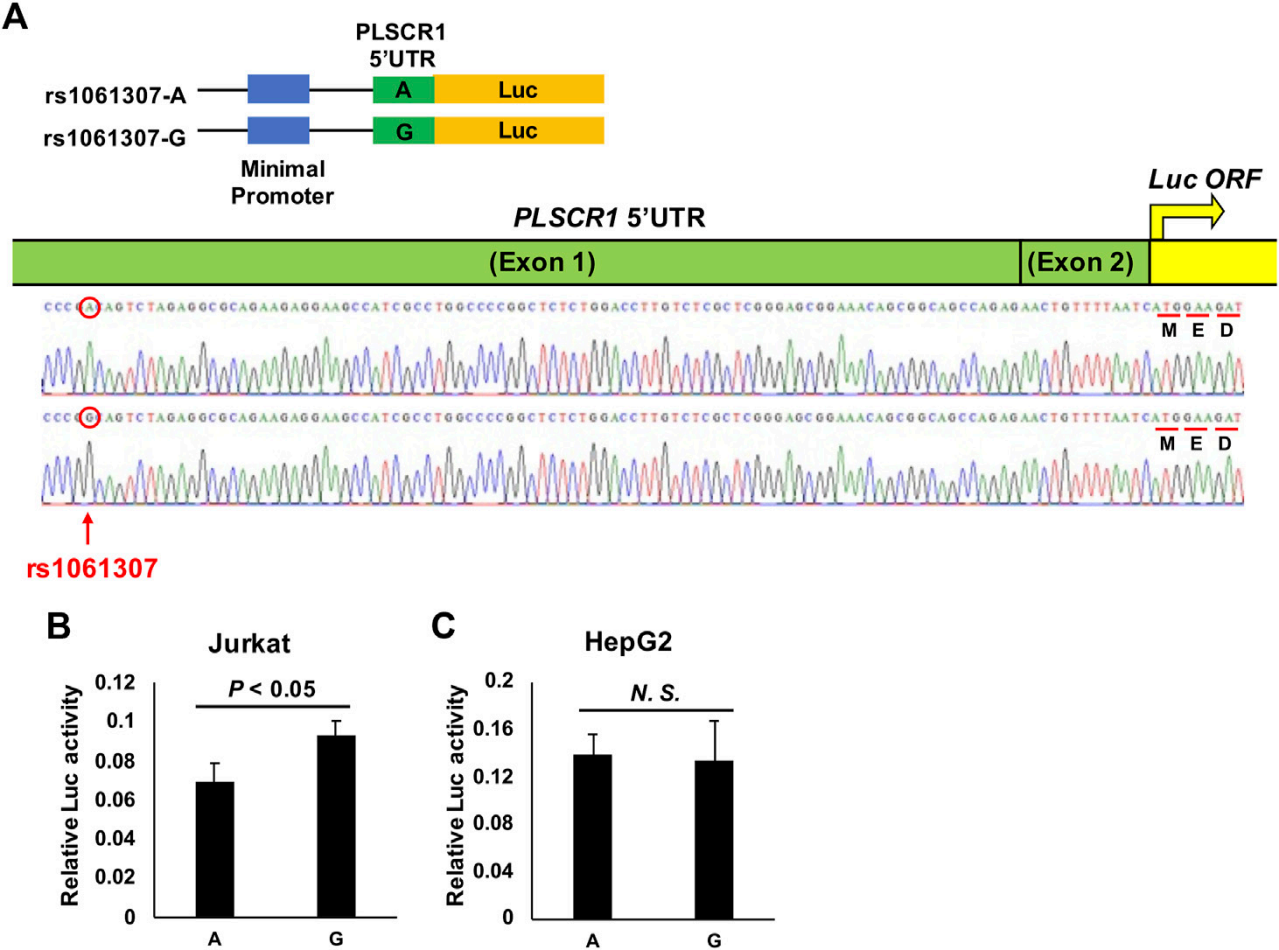
Luciferase assay for *PLSCR1* rs1061307. **(A)** Construct of the luciferase reporter pGL4.23 (luc2/minP) vector. *PLSCR1* 5′UTR was conjugated with luc ORF. The transcriptional enhancing activity of these plasmid constructs was measured by assaying the luciferase (luc) activity of the transfected Jurkat **(B)** and HepG2 **(C)** cells, 24 h after transfection. The values of relative luciferase activity are shown as mean ± SD.

The *PLSCR1* gene was previously observed to be associated with the inhibition of HBV replication (Yang et al., 2012) *in vitro* and *in vivo*; thus, in this study, we decided to conduct a functional assay to identify the primary functional variant for this locus. Among the top 100 susceptibility SNPs in the *PLSCR1* locus, rs1061307 was the only one located in genomic regions linked to histone acetylation, such as H3K27Ac and DNase I hypersensitivity, in at least one cell type in the UCSC genome browser (http://genome.ucsc.edu/index.html) (Kent et al., 2002) (Supplementary Figure S4). This SNP was selected as a potential candidate for disease susceptibility through regulation of the level of *PLSCR1* gene expression. We conducted a luciferase assay (Figure 3) to evaluate the regulation of *PLSCR1* gene expression by rs1061307 using the Jurkat cells (human T lymphocytes) and HepG2 cells (human liver carcinoma cells). Twenty-four hours after transfection of the pGL4.23 constructs into Jurkat cells, the luciferase activity was significantly higher with the G allele than the A allele of rs1061307 (*p*-value <0.05) (Figure 3B). This tendency was concordant with the e-QTL data of rs1061307 on endogenous *PLSCR1* gene expression in all tissues registered in the GTEx portal database (http://gtexportal.org/home/) (Lonsdale et al., 2013), with the most significant association observed in whole blood cells (Supplementary Figure S5). This result suggested that rs1061307 was the primary functional variant for *PLSCR1* locus. However, no difference in luciferase activity between the rs1061307 G and A alleles was observed in HepG2 cells (Figure 3C) which is likely due to differences in the binding of transcription factors to these alleles in different cell types.

## Discussion

In the current study, the most significant association was detected between the *HLA* class II region and HBV chronicity in the Thai population, replicating the results of previous studies. In addition, we observed the suggestive association of several novel candidate loci, including the *PLSCR1* gene on chromosome 3, the *PDLIM5* gene on chromosome 4, the *SGPL1* gene on chromosome 10, and the *MGST1* gene on chromosome 12. We also conducted a luciferase assay, and identified rs1061307 as the primary functional variant for the *PLSCR1* gene.

In the current study, we identified the novel association of rs35766154 (*PLSCR1* gene located on chromosome 3, *p*-value = 7.39 × 10^−7^, OR = 0.43, 95% CI = 0.31–0.61) with HBV chronicity. The *PLSCR1* gene encodes PLSCR1, an α/β interferon (IFN)-inducible protein that has been shown to mediate antiviral activity against RNA viruses, such as vesicular stomatitis virus and encephalomyocarditis virus, through the enhancement of IFN responses (Dong et al., 2004). PLSCR1 was also observed to inhibit the replication of HBV *in vivo* and *in vitro*, as well as in a mouse model, through a significant decrease in HBV RNA and DNA intermediates and proteins (Yang et al., 2012), the activation of the Jak/Stat pathway (Yang et al., 2012), enhancement of IFN responses (Li Q. et al., 2016), and regulation of HBV-encoded X protein (HBx) stability (Yuan et al., 2015). *In silico* gene expression analysis of the *PLSCR1* locus in the GTEx database (http://gtexportal.org/home/) (Lonsdale et al., 2013) showed that SNP rs1061307 was correlated with changes in *PLSCR1* gene expression level in several tissues, with the most significant association observed in whole blood (*p*-value = 1.8 × 10^−22^). The *PLSCR1* gene has several isoforms, and SNP rs1061307 is located in the promoter region of some of the isoforms. We further confirmed the significant association of rs1061307 alleles with *PLSCR1* gene expression in Jurkat cells. This result suggested that rs1061307 is the primary functional variant for the *PLSCR1* locus, affecting the risk of HBV chronicity in the Thai population.

There were three more suggestive associations observed on chromosomes 4, 10, and 12. Although further mapping and functional studies are needed to confirm these associations, there are some biologically plausible explanations. The SNP with the lowest *p*-value on chromosome 4 was rs62321986, which is located 28 kb 5’ of the *PDLIM5* gene. This gene encodes Enigma Homolog 1 (ENH1, PDLIM5), a PDZ-LIM protein that interacts with and activates protein kinase C via the direct binding of its LIM domain (Kuroda et al., 1996; Maturana et al., 2011). Protein kinase C, among other protein kinases, phosphorylates HBV core protein, which modulates HBV replication at multiple stages (Kann and Gerlich, 1994; Jung et al., 2014). Another significant association was observed on chromosome 10 for SNP rs144998273 which is located in the intron of the *SGPL1* gene. Based on the GTEx portal database (http://gtexportal.org/home/) (Lonsdale et al., 2013), different genotypes of rs144998273 are associated with different expression levels of the *SGPL1* and *PCBD1* (Pterin-4 Alpha-Carbinolamine Dehydratase 1) genes. The *SGPL1* gene encodes sphingosine-1-phosphate lyase 1, which catalyzes the final degradative step in the sphingolipid metabolic pathway (Fyrst and Saba, 2008). Sphingolipids play important roles in several aspects of the HBV life cycle, and blockage of sphingolipid biosynthesis, which results in the inhibition of HBV replication, was suggested to be a potential therapeutic strategy for hepatitis B (Tatematsu et al., 2011). The *PCBD1* gene encodes pterin-4 alpha-carbinolamine dehydratase, which is a dimerization cofactor for the transcription factor hepatocyte nuclear factor 1α (HNF1α), and it enhances the transcription activity of HNF1α (Sourdive et al., 1997). HNF1α downregulates the expression of HBV genes and viral replication by activating the NF-κB signaling pathway (Lin et al., 2017). The last non-*HLA* suggestive association in this study was observed with SNP rs1828682, which is located in the intron of the *MGST1* gene on chromosome 12. This gene encodes Microsomal Glutathione S-Transferase 1, which is an important enzyme for redox control and detoxification (Morgenstern et al., 2011). It has been observed that HBV causes oxidative stress, which leads to liver carcinogenesis and cell death (Shirakata and Koike, 2003; Hu et al., 2011). Further functional studies and replication analyses in the Thai population as well as in other populations are required to confirm the associations detected in the current study.

The top SNPs of the *HLA* region reported by previous GWASs, such as rs3077 (*HLA-DPA1*) (Kamatani et al., 2009; Mbarek et al., 2011; Nishida et al., 2012), rs9277535 (*HLA-DPB1*) (Kamatani et al., 2009; Mbarek et al., 2011; Nishida et al., 2012), rs7453920 (*HLA-DQ*) (Mbarek et al., 2011), and rs2856718 (*HLA-DQ*) (Mbarek et al., 2011), were replicated in the current study, but not as the lead SNP. The GWAS top hit in the *HLA* region was rs7770370 (*p*-value = 7.71 × 10^−10^, OR = 0.49, 95% CI = 0.39–0.61). This SNP was reported as the lead SNP in two GWASs of chronic HBV infection and responsiveness to HBV vaccination (Wu et al., 2015; Huang et al., 2020). A positive correlation between the rs7770370 A allele and *HLA-DPB1* protective alleles DPB1*02:01, *02:02*, *03:01*, *04:01*, and *14:01* (*r*: 0.26–0.76; all *p*-values < 10^–5^) and a negative correlation between the rs7770370 A allele and *HLA-DPB1* risk allele DPB1*05:01 (*r*: −0.76; *p*-values < 10^–50^) have been reported in a previous study (Wu et al., 2015). The high linkage between the rs7770370 A allele and *HLA-DPB1* protective alleles might partly explain the protective effect of the SNP observed in the current study.

The important role of *HLA* class II genes in the chronicity of HBV infection has been extensively examined in different ethnicities (Singh et al., 2007; Kamatani et al., 2009; Nishida et al., 2014, 2015; Zhu et al., 2016). Here, we used a highly accurate *HLA* imputation method, and showed the association of several *HLA* alleles with chronic HBV infection in the Thai population. Our results revealed the significant association of HLA-DPA1*02:02, HLA-DPB1*05:01, HLA-DPB1*13:01, and HLA-DQB1*03:03 with susceptibility to HBV chronicity, and the significant association of HLA-DPA1*01:03, HLA-DPB1*02:01, and HLA-DQB1*06:09 with protection against HBV chronicity. HLA-DPA1*01:03 and HLA-DPB1*13:01 were previously shown to be significantly associated with chronic hepatitis B in the Thai population (Nishida et al., 2014) with protective and susceptibility effects, respectively. The HLA-DQB1*06:09 and *HLA-DPA1/DPB1* alleles which we reported here, have been detected to be associated with chronic hepatitis B with similar tendencies in previous reports in different ethnic groups (Cho et al., 2008; Kamatani et al., 2009; Hu et al., 2013; Nishida et al., 2014, 2015, 2016; Li Y. et al., 2016; Zhu et al., 2016; Ou et al., 2019, 2021; Huang et al., 2020). However, HLA-DQB1*03:03 is associated with both an increased (Mbarek et al., 2011) and decreased risk of chronic hepatitis B (Xi-Lin et al., 2006; Zhang et al., 2015; Huang et al., 2016). No locus other than *HLA-DPA1/DPB1* showed a significant independent association with HBV persistence after applying the regression analysis with the GWAS top hit as the covariate. However, the significant association of two alleles from the *HLA-DQ* locus (HLA-DQB1*03:03 and HLA-DQB1*06:09) was detected after *HLA* allele imputation. This shows the necessity of analyzing *HLA* alleles independently, because they may not be captured by GWAS arrays.

We also detected the association of two- and three-locus *HLA* haplotypes with HBV chronicity, including HLA-DPA1*01:03-DPB1*02:01 with protective effect, and HLA-DPA1*02:02-DPB1*05:01, HLA-DPB1*05:01-DQB1*03:03, HLA-DPB1*13:01-DQB1*05:02, and HLA-DPA1*02:02-DPB1*05:01-DQB1*03:03 with a susceptibility effect. The protective effect of HLA-DPA1*01:03-DPB1*02:01 against chronic hepatitis B was reported by Nishida et al. (Nishida et al., 2014). Although the association of HLA-DPB1*13:01 with susceptibility to HBV chronicity in Thai subjects was shown in the study by Nishida et al. (Nishida et al., 2014), no significant association of this allele was observed in any *HLA-DPA1-DPB1* haplotypes in their study, which is consistent with our results. However, we detected the significant association of HLA-DPB1*13:01 in an *HLA-DPB1-DQB1* haplotype (HLA-DPB1*13:01-DQB1*05:02), which again emphasizes the necessity of analyzing *HLA* alleles independently of *HLA* loci detected in GWASs. Additionally, HLA-DPB1*05:01 and three different haplotypes bearing this allele (HLA-DPA1*02:02-DPB1*05:01, HLA-DPB1*05:01-DQB1*03:03, and HLA-DPA1*02:02-DPB1*05:01-DQB1*03:03), were found to be significantly associated with susceptibility to chronic HBV infection in our study, but they were not observed to be associated with HBV chronicity in the Thai subjects in the study by Nishida et al. (Nishida et al., 2014); this discrepancy may be due to the relatively small number of healthy control subjects in their study. Two studies (Kamatani et al., 2009; Nishida et al., 2014) have reported HLA-DPA1*02:02-DPB1*05:01 as a susceptibility factor for HBV chronicity in the Japanese population. The consistency of our results with those of previous reports indicates the reliability of our findings as well as the advantage of *HLA* imputation for identifying disease-associated *HLA* alleles and loci.

There are some limitations to the current study. This study suffers from a limited sample size and lack of a replication sample set from the Thai population to validate the candidate associations detected in the GWAS stage in an independent cohort. However, we replicated the previously reported association of the *HLA* class II region with HBV persistence, which confirmed the reliability of our results in this cohort. In addition, the control subjects used in this study were healthy controls without previous exposure to HBV; they remain at the risk of being exposed to the virus and acquiring persistent infection, which may have limited our ability to compare the polymorphisms to those in patients with chronic HBV infection. To address these issues, larger sample sets from the Thai population and control subjects that have previously been exposed to HBV should be examined.

## Conclusion

This study is the first GWAS of HBV chronicity in the Thai population. With the limitations discussed above, such as the limited sample size and lack of an independent replication sample set from the Thai population, *PLSCR1* was detected as a potential genetic locus for chronic hepatitis B with plausible biological roles against HBV. Additionally, other loci, including *PDLIM5* (rs62321986) and *SGPL1* (rs144998273), were identified as susceptibility candidate loci for persistent HBV infection, and *MGST1* (rs1828682) was identified as a protective candidate locus against persistent HBV infection. However, further research and functional studies in independent cohorts are strongly recommended. The observations of the current study indicate the necessity of conducting genomic research in diverse populations for the same disease, as differences in disease prevalence along with differences in allele frequencies and variant effect sizes in different populations may lead to detection of novel variants underlying the pathogenicity of the disease.

## Materials and Methods

### Samples

This study included 329 patients with chronic HBV infection who were recruited at Chulalongkorn University in Bangkok, Thailand, and 318 healthy control subjects who were blood donors at the National Blood Center, The Thai Red Cross Society, in Bangkok, Thailand. The diagnosis of chronic HBV infection was established according to the guideline for the diagnosis and treatment of chronic hepatitis B based on HBsAg seropositivity and elevated serum alanine aminotransferase levels (>1.5 times the upper limit of normal [35 IU/L]) for a period of longer than 6 months (with at least three bimonthly tests). Among the 329 patients, 100 were diagnosed with hepatocellular carcinoma, and 77 with liver cirrhosis. All healthy controls were seronegative for HBsAg and hepatitis B core antibody.

### Genome-Wide SNP Genotyping and Genotype Calling

All samples were genotyped using the Axiom Genome-Wide ASI 1 Array (Thermo Fisher Scientific, Inc., Waltham, MA). Genotyping steps were performed according to the manufacturer’s protocol. The final data were produced as CEL files (intensity files). We conducted genotype calling for 600,014 SNPs using Affymetrix Genotyping Console software (v4.2.0.26). The Axiom GT1 algorithm was used for generating the genotype data. All samples had Dish QC > 0.82. One case sample with a genotype calling rate <0.97 was omitted. We then leveraged the Ps_Classification functionality of SNPolisher in Analysis Power Tools (Affymetrix, 2022) to calculate SNPs QC metrics. SNPs with good genotype quality were included in the list of recommended SNPs for downstream analysis. SNPs not included in the list were subsequently excluded from the data. SNP & Variation Suite software (Golden Helix Inc., Bozeman, MT, USA) was used to convert the results of genotype calling to input files for PLINK (v1.9) (Purcell et al., 2007).

### IBD and PCA

Before conducting IBD, we performed linkage disequilibrium-based SNP pruning using PLINK (v1.9) (Purcell et al., 2007) with the option “--indep-pairwise 50 5 0.5”. We then excluded the SNPs in the prune.out output file, and conducted IBD using the options “--genome” and “--min 0.05”. One sample from each pair of samples with PI_HAT >0.1875 (halfway between the expected IBD for second- and third-degree relatives (Anderson et al., 2010)) was removed from the data.

For PCA, we downloaded the HapMap phase III data from the HapMap homepage (https://www.sanger.ac.uk/resources/downloads/human/hapmap3.html). We merged the study data with four populations from the public HapMap data, i.e., Yoruba in Ibadan, Nigeria (YRI), Utah Residents (CEPH) with Northern and Western European Ancestry (CEU), Han Chinese in Beijing, China (CHB), and Japanese in Tokyo, Japan (JPT). QC was conducted on the data using PLINK (v1.9) (Purcell et al., 2007) with the options “--geno 0.03”, “--maf 0.01”, and “--hwe 0.000001”. Then, grm files were produced by GCTA software (Yang et al., 2011) using the option “--make-grm”. PCA was conducted by GCTA software (Yang et al., 2011) using the option “--pca 10”.

### Whole-Genome Imputation

We performed pre-imputation QC and excluded SNPs with a MAF <0.01 and call rate <0.97 from all samples, and a HWE *p*-value <1 × 10^−6^ in healthy controls. The SNPs that passed the QC were pre-phased with 1,000 Genomes phase III as the reference data using SHAPEIT software (v2.17) (Delaneau et al., 2012). Whole-genome imputation of pre-phased data was conducted with 1,000 Genomes phase III as the reference panel using IMPUTE2 software (v2.3.2) (Howie et al., 2009) on 7-Mbp regions with the options “-Ne 2000”, “-k_hap 1,000”, “-k 120”, “-burnin 15”, and “-iter 50”. After imputation, SNPs with an info metric ≥0.8 were extracted.

### Association Analysis

For the association analysis, PLINK (v1.9) (Purcell et al., 2007) was used with the options “--assoc”, “--adjust qq-plot”, and “--ci 0.95”.

### Validation of the Association Results Using the TaqMan Assay

The TaqMan assay (Breen et al., 2000), which is a robust, accurate, cost-effective, and high-throughput technique for the discrimination of alleles differing by a single base change, was used in the current study for the validation of candidate SNPs. We conducted genotyping assays using made-to-order probes (Thermo Fisher Scientific, Waltham, MA) and the KAPA Probe Fast Universal qPCR Kit master mix (Kapa Biosystems, Boston, MA) according to the manufacturer’s instructions. The fluorescence intensity data from genotyping assays were converted into genotype calls by the Roche LightCycler 480 system (Roshe, Basal, Switzerland). The consistency rate of the genotype calls of candidate SNPs was calculated by comparison to those from the GWAS array.

### *HLA* Typing

*HLA* typing was conducted using LABType SSO *HLA* DPA1/DPB1 kit (One Lambda, CA) and a Luminex Multi-Analyte Profiling system (xMAP; Luminex, Austin, TX) according to the manufacturer’s protocol. The genotyping results were compared to the imputation data and concordance rates were calculated.

### Luciferase Assay

Specific polymerase chain reaction primers (Supplementary Table S10) were used to amplify the 5′UTR sequence of the *PLSCR1* gene, including each allele of rs1061307 from human genomic DNA. Then, a NEBuilder HiFi DNA Assembly kit (New England Biolabs, Ipswich, MA) was used for the subcloning of the amplicons into the luciferase reporter pGL4.23 (luc2/minP) vector (Promega, Madison, WI) in the Nco I site (located in the translation start site of the luciferase gene). Lipofectamine 3,000 (Thermo-Fisher Scientific, Waltham, MA) was used to transfect pGL4.23 constructs of each allele (1 μg) and the pGL4.74 (hRluc/TK) vector (100 ng), which was used as an internal control, into the Jurkat and HepG2 cells. The Dual-Luciferase Reporter Assay System (Promega, Madison, WI) was used for measuring the luciferase activity. The relative luciferase activity of the major and minor alleles of rs1061307 was compared using Student’s t-test. *p*-values < 0.05 were regarded to be statistically significant.

## Data Availability

The GWAS data presented in the current study are deposited in the public database “NBDC Human Database”, accession number “hum0075.v3.Thai-gwas.v1”.
