# Supplementary material for: Genome-Wide Association Study for Chronic Hepatitis B Infection in the Thai Population
Source: Front Genet. 2022 Jun 13;13:887121. doi: 10.3389/fgene.2022.887121 (PMC9234442; doi:10.3389/fgene.2022.887121)
Supplement: Supplementary file 1 [file DataSheet1.docx]

**a.**  **b.**


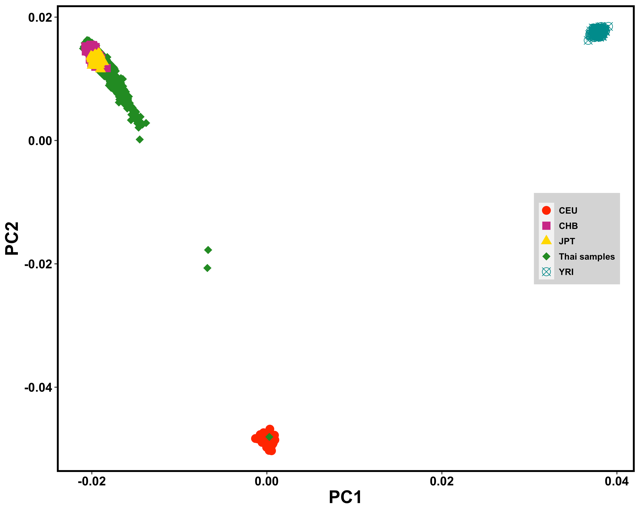

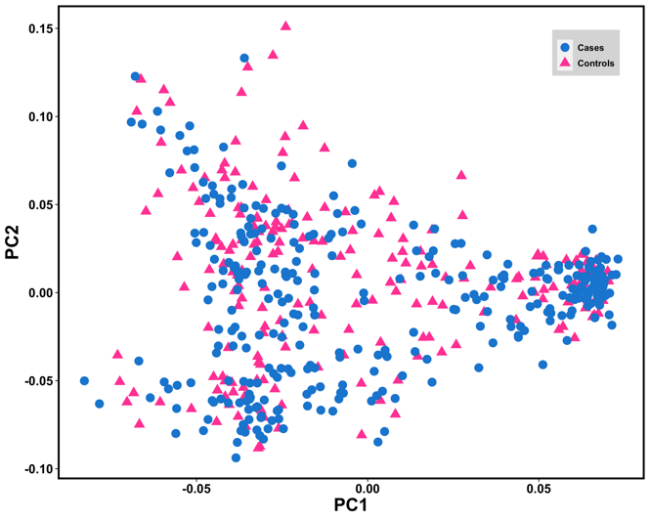


**Fig. S1. Principal component analysis (PCA) of GWAS stage.** Figure a. shows the PCA plot of chronic hepatitis B cases and controls from Thai population with HapMap samples (JPT, CHB, YRI, and CEU samples). Thai samples clustered with Asian (JPT and CHB) samples. Three samples (shown with red arrows on the figure) were detected as population outliers and excluded from the data. Figure b. shows the PCA plot of chronic hepatitis B cases and controls from Thai population only. Cases and controls shared similar genetic profiles.

**a.**


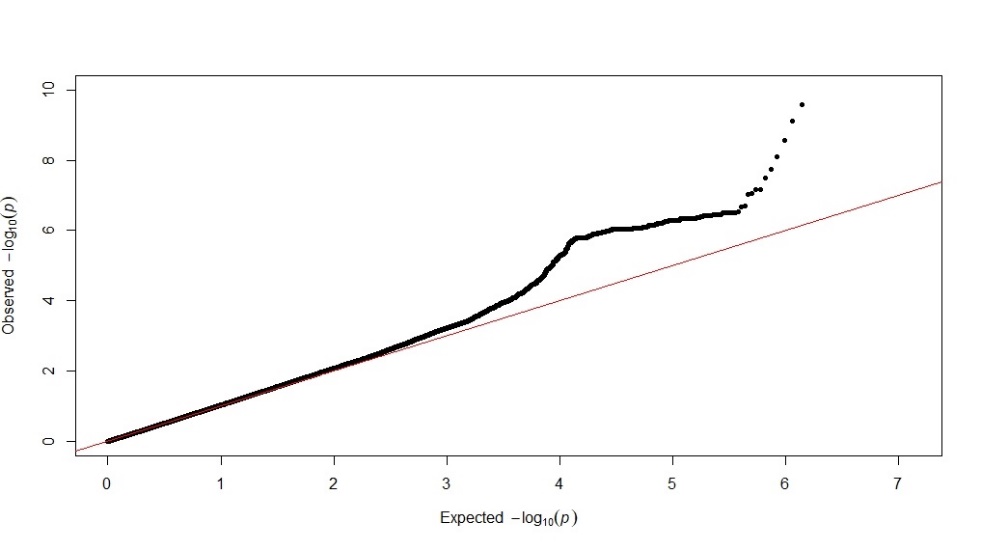


**b.**


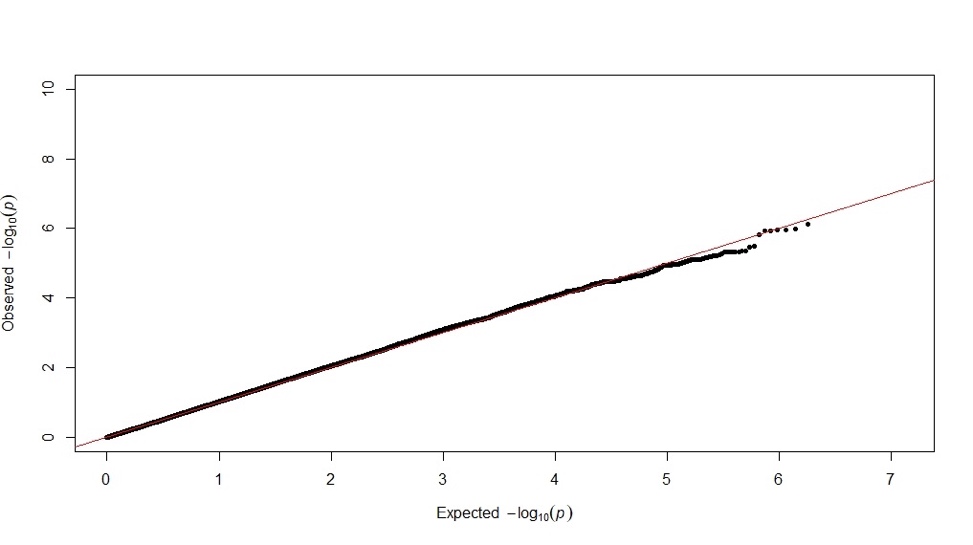


**Fig. S2:** **Quantil-quantile plots for P-values of each SNP calculated from chi-square test in GWAS.** The inflation factor (λ_GC_) was estimated to be **(a.)** 1.036 for all tested SNPs including those located in the *HLA* region, and **(b.)** 1.031 when SNPs located in the *HLA* region (hg19: chr6:25,652,429-33,368,333) were excluded.

1. **b.**

**
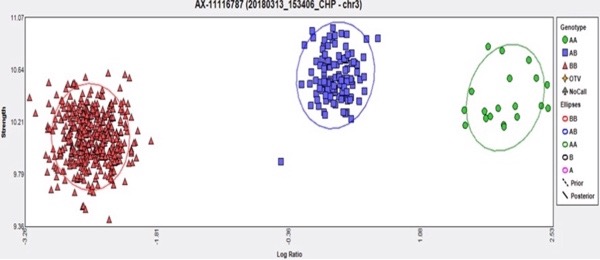
**
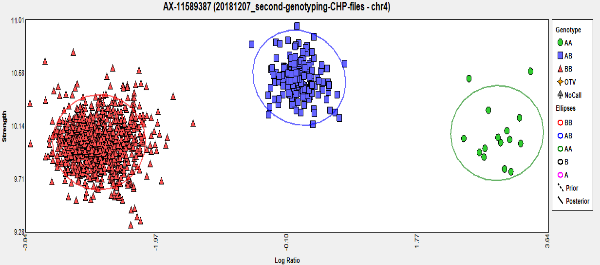


**c. d.**
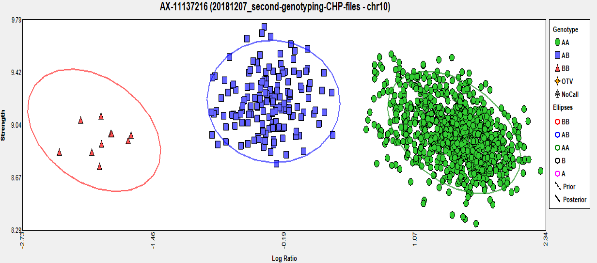

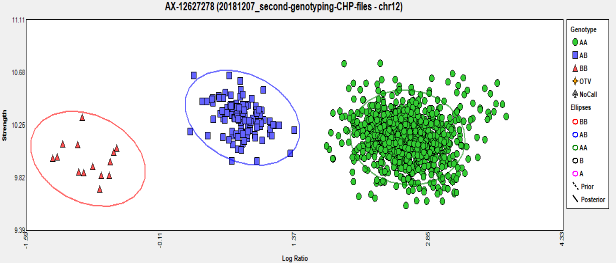


**e.** **f.**

**
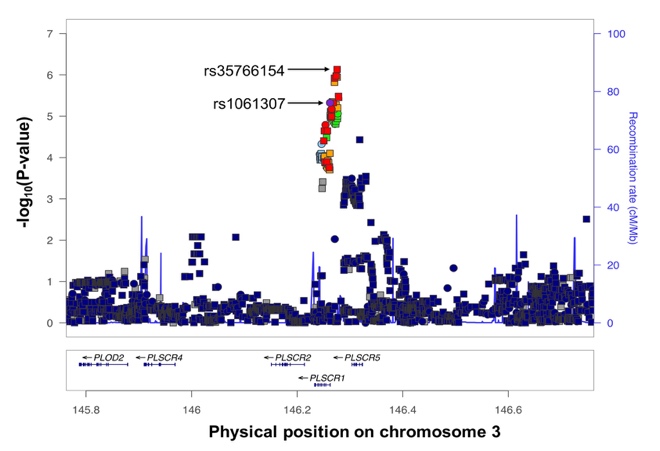
**
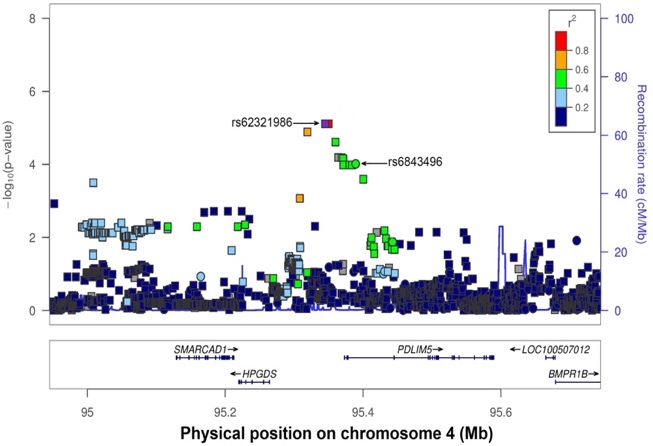


**g. h.**

**
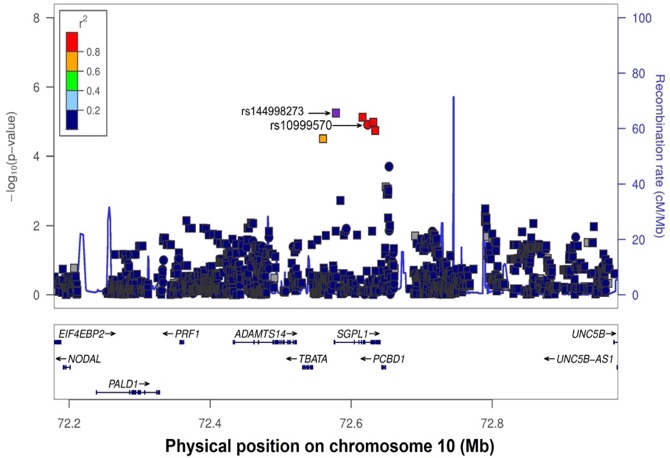

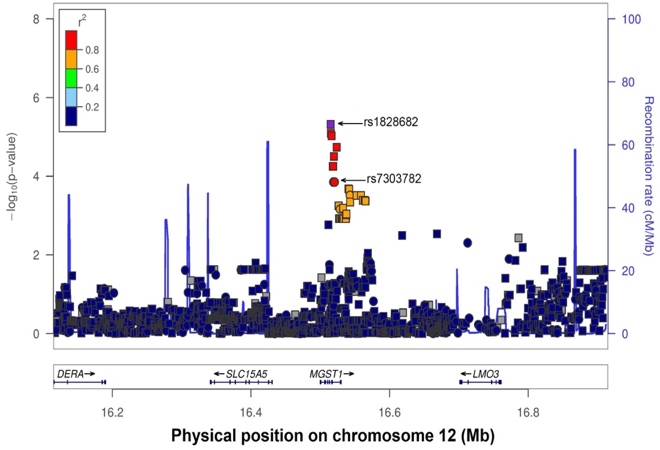
**

1. **j.**

**
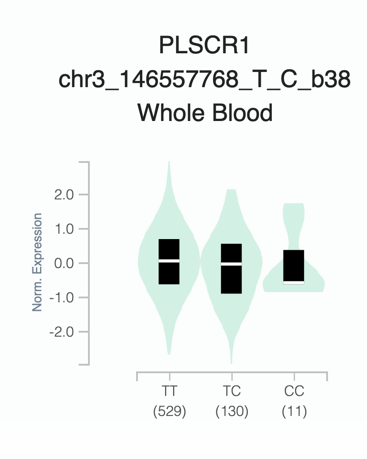

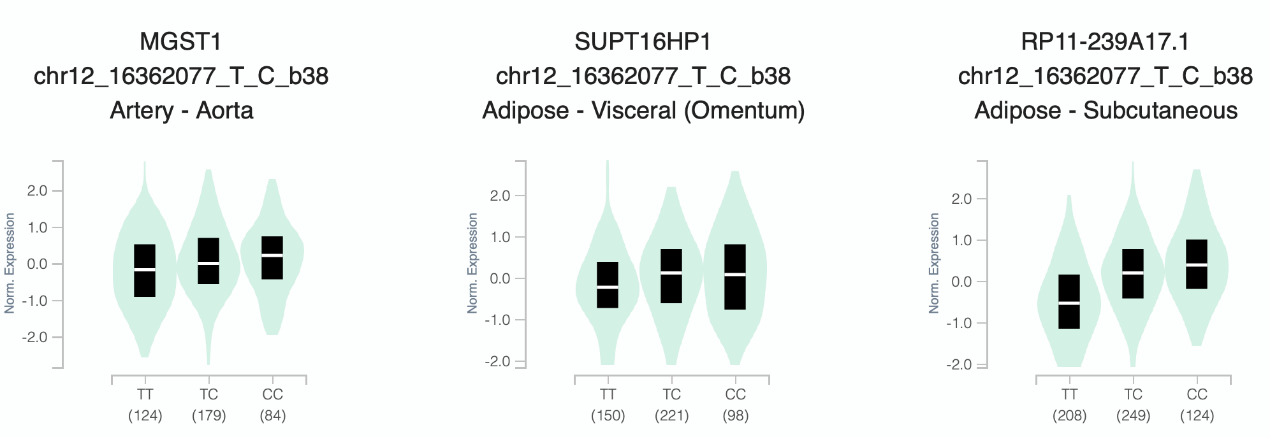
**

NES = 0.14, p-value = 1.1 × 10^-5^

NES = -0.12, p-value = 3.0 × 10^-6^

**k.**

**
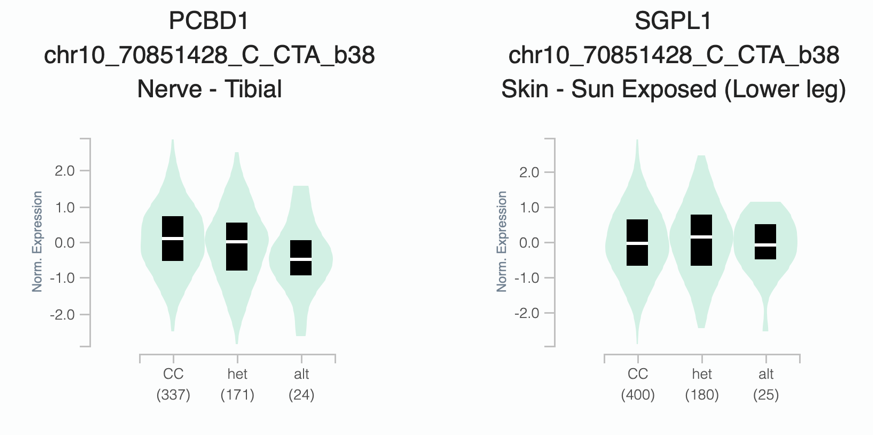
**

NES = -0.20, p-value = 2.7 × 10^-13^  NES = 0.11, p-value = 5.7 × 10^-10^

**Fig. S3: Plots for loci detected to be associated with chronic hepatitis B with suggestive level of significance.** Figures a., b., c., and d. show the genotype cluster plots for the most significant genotyped SNP from each locus: rs1061307 on chromosome 3, rs6843496 on chromosome 4, rs10999570 on chromosome 10, and rs7303782 on chromosome 12, respectively. Figures e., f., g., and h. show the regional association plot for the suggestive associations observed on chromosomes 3, 4, 10, and 12. Imputed SNPs are shown using squares and genotyped ones are shown using circles. The most significant imputed SNP and the most significant genotyped SNP are shown with arrows. Figure i. shows the endogenous expression of *PLSCR1* gene in the Whole Blood among different people with different genotypes of the SNP rs35766154. Figure j. shows the endogenous expression of *MGST1* gene in Artery – Aorta organ among different people with different genotypes of the SNP rs1828682 (the most significant SNP on chromosome 12). Figure k. shows the endogenous expression of *PCBD1* and *SGPL1* genes in the Nerve – Tibial and skin (sun exposed) organs respectively among different people with different genotypes of the SNP rs144998273 (the most significant SNP on chromosome 10). The endogenous expressions of genes were extracted from the GTEx portal database (<http://gtexportal.org/home/>). There was no gene expression data reported for the most significant SNP on chromosome 4 (rs62321986) in GTEx portal database.


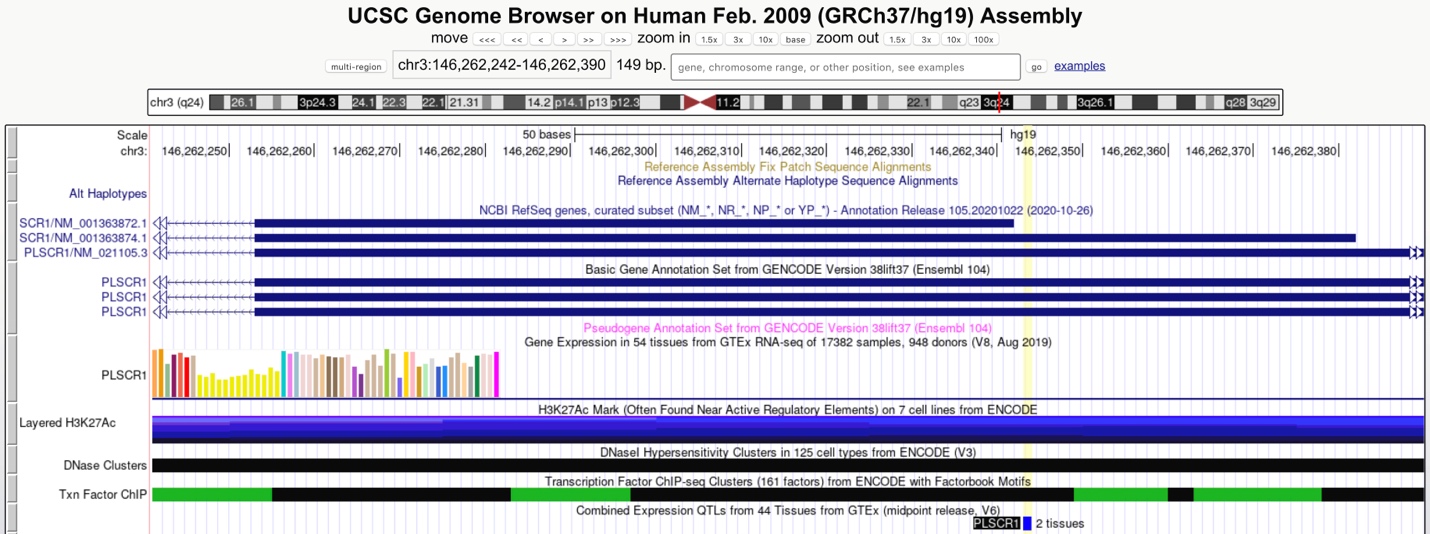


**Fig. S4: Selection of the candidate primary variant for functional analysis.** Among the top 100 SNPs at *PLSCR1* locus, rs1061307 was selected as the final candidate using the UCSC genome browser (http://genome.ucsc.edu/index.html) as it was found to be located in transcription regulatory elements defined by (1) DNase I hyper-sensitivity cluster analysis of any one of 125 cell types and (2) the existence of H3K27Ac markers in any one of the cell lines


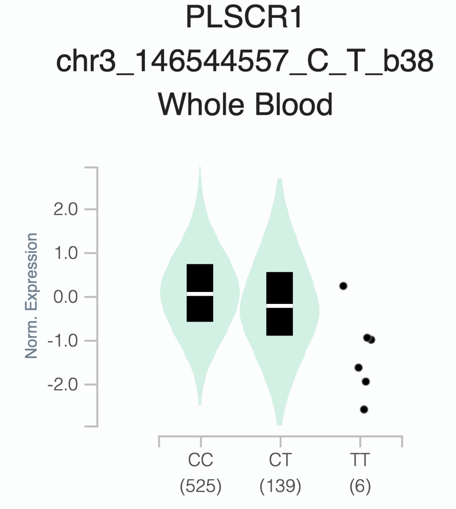


NES = -0.25, p-value = 1.8 × 10^-22^

**Fig. S5: The endogenous expression of *PLSCR1* gene in whole blood cells.** The violin plot shows different expression levels of *PLSCR1* gene among people with different genotypes of rs1061307. The data of endogenous *PLSCR1* gene expressions were extracted from the GTEx portal database (<http://gtexportal.org/home/>).
